# Risk factors for *Klebsiella pneumoniae* carbapenemase (KPC) gene acquisition and clinical outcomes across multiple bacterial species

**DOI:** 10.1016/j.jhin.2020.01.005

**Published:** 2020-04

**Authors:** A.J. Mathers, K. Vegesana, I. German-Mesner, J. Ainsworth, A. Pannone, D.W. Crook, C.D. Sifri, A. Sheppard, N. Stoesser, T. Peto, A.S. Walker, D.W. Eyre

**Affiliations:** a Division of Infectious Disease and International Health, Department of Medicine, University of Virginia Health System, Charlottesville, VA, USA; b Clinical Microbiology Laboratory, Department of Pathology, University of Virginia Health System, Charlottesville, VA, USA; c Health Information and Technology, University of Virginia Health System, Charlottesville, VA, USA; d Department of Public Health Sciences, University of Virginia, School of Medicine, Charlottesville, VA, USA; e Nuffield Department of Clinical Medicine, University of Oxford, Oxford, UK; f National Institutes of Health Research, Health Protection Research Unit in Healthcare Associated Infection and Antimicrobial Resistance, University of Oxford, Oxford, UK; g Office of Hospital Epidemiology, University of Virginia Health System, Charlottesville, VA, USA; h Big Data Institute, University of Oxford, Oxford, UK

**Keywords:** Carbapenemase-producing Enterobacterales (CPE), Carbapenemase-producing organisms (CPO), *Klebsiella pneumoniae* carbapenemase (KPC), Multi-species clinical risk, Carbapenem-resistant Enterobacterales (CRE)

## Abstract

**Introduction:**

Risk factors for carbapenemase-producing Enterobacterales (CPE) acquisition/infection and associated clinical outcomes have been evaluated in the context of clonal, species-specific outbreaks. Equivalent analyses for complex, multi-species outbreaks, which are increasingly common, are lacking.

**Methods:**

Between December 2010 and January 2017, a case–control study of *Klebsiella pneumoniae* carbapenemase (KPC)-producing organism (KPCO) acquisition was undertaken using electronic health records from inpatients in a US academic medical centre and long-term acute care hospital (LTACH) with ongoing multi-species KPCO transmission despite a robust CPE screening programme. Cases had a first KPCO-positive culture >48 h after admission, and included colonizations and infections (defined by clinical records). Controls had at least two negative perirectal screens and no positive cultures. Risk factors for KPCO acquisition, first infection following acquisition, and 14-day mortality following each episode of infection were identified using multi-variable logistic regression.

**Results:**

In 303 cases (89 with at least one infection) and 5929 controls, risk factors for KPCO acquisition included: longer inpatient stay, transfusion, complex thoracic pathology, mechanical ventilation, dialysis, and exposure to carbapenems and β-lactam/β-lactamase inhibitors. Exposure to other KPCO-colonized patients was only a risk factor for acquisition in a single unit, suggesting that direct patient-to-patient transmission did not play a major role. There were 15 species of KPCO; 61 (20%) cases were colonized/infected with more than one species. Fourteen-day mortality following non-urinary KPCO infection was 20% (20/97 episodes) and was associated with failure to achieve source control.

**Conclusions:**

Healthcare exposures, antimicrobials and invasive procedures increased the risk of KPCO colonization/infection, suggesting potential targets for infection control interventions in multi-species outbreaks. Evidence for patient-to-patient transmission was limited.

## Introduction

Carbapenemase-producing Enterobacterales (CPE) remain one of the most urgent healthcare threats. Several Enterobacterales spp., such as *Escherichia coli* and *Klebsiella pneumoniae*, are common human pathogens and asymptomatic colonizers of the human gastrointestinal tract and environmental niches. Others species such as *Kluyvera intermedia* are more adapted to environmental reservoirs, but may play an important role in resistance gene exchange and dissemination in both healthcare and non-healthcare settings [1]. Clinically significant carbapenem resistance occurs across Enterobacterales spp., particularly *Klebsiella* spp., *E. coli* and *Enterobacter* spp. [[2], [3], [4]], and is most often mediated by carbapenemase genes which can be shared across species. *K. pneumoniae* carbapenemase (KPC, encoded by *bla*_KPC_) is one of the most common carbapenemase genes globally [5].

Existing guidelines for CPE management [6] have largely been based on evidence from clonal, single-species outbreaks, with a view that patient-to-patient spread has played a key role and colonized patients represent a major risk [7,8]. Multiple co-morbidities, antimicrobial exposure, critical illness and exposure to other colonized patients are risk factors for acquisition and infection [[7], [8], [9], [10]]. There is increasing recognition, however, that CPE outbreaks are evolving into complex, multi-species, polyclonal phenomena, facilitated by the rapid horizontal transmission of carbapenem resistance genes on mobile genetic elements such as plasmids [2,11]. In these contexts, the healthcare environment, and wastewater reservoirs in particular, may play a major role in transmission [12,13]. Particular clinical risk factors for acquisition in these contexts remain poorly defined, partly because robust screening programmes for asymptomatic colonization with all species of CPE are not widely implemented [14].

In the study setting, endemic transmission of multi-species KPC-producing organisms (KPCO) has occurred since 2007 despite robust patient surveillance. The wastewater environment likely played a role in transmission [15]. This provides a unique opportunity to systematically examine risk factors associated with: (i) multi-species KPCO acquisition; (ii) KPCO infection vs colonization; and (iii) 14-day mortality in those with KPCO infection. This approach allowed the authors to investigate which patients were at risk of acquisition of KPCO colonization, and then, from the subset of patients who became colonized, to identify which patients were at risk of invasive infection, as, whilst acquisition is generally considered to precede invasion (even if this is short-lived), the drivers for these two processes (acquisition without invasion vs invasion) may be distinct.

## Methods

### Setting and samples

The University of Virginia Health System (UVaHS) consists of a 619-bed academic tertiary acute care hospital and a 44-bed long-term acute care hospital (LTACH) (opened in 2012). During the study (1^st^ December 2010–1^st^ January 2017), admission and weekly perirectal KPCO screening was performed on all patients admitted to the LTACH, the surgical trauma burn (STBICU) and medical (MICU) intensive care units, and anywhere another inpatient on any ward had been identified as colonized or infected with KPCO (until 7 days after the last KPCO case), using methods described previously (detailed laboratory and screening methods in online supplementary material) [11].

### KPCO acquisition

**Figure 1:**
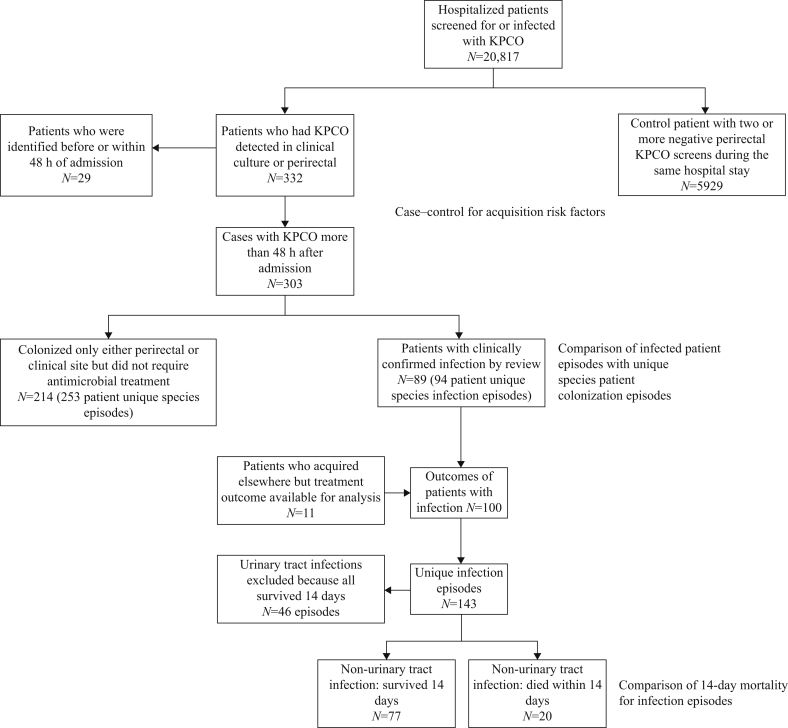
Study flow chart. KPCO, *Klebsiella pneumoniae* carbapenase-producing organisms.

Risk factors for KPCO acquisition were identified using a case–control study, including patients who spent >48 h within the acute care hospital or LTACH during the study period. Cases were defined as any patient whose first KPCO-positive culture (either from screening or clinical samples, deemed an ‘acquisition’) was taken >48 h after their first admission to the institution, to minimize inclusion of imported KPCO cases whose risk of acquisition would be difficult to ascertain (Figure 1). Controls had no positive cultures and two consecutive negative perirectal cultures within the same hospital stay (mostly ≥7 days apart due to the screening policy) to minimize the impact of false-negative rectal screens.

Potential risk factors were obtained from an infection control data warehouse of electronic medical records, including patient location, length of acute care hospital stay and any LTACH stays, procedure and diagnostic codes, medication exposures, and microbiology results (see online supplementary material for details). Exposures were determined for inpatient events during the 90 days preceding the first KPCO-positive culture for cases and prior to the last negative screen for controls. Total event counts during the 90 days were considered for recurring exposures in inpatients [e.g. days of enteral feeding or patient-days of KPCO colonization pressure arising from sharing a unit with at least one KPCO-positive patient and indicating potential for direct patient-to-patient transmission (see online supplementary material for calculation)]. KPCO colonization pressure was considered as a separate predictor for each ICU, other units in the acute care hospital and the LTACH as the screening strategies differed in each location, and in the case of other units, screening was triggered by identification of a colonized patient, thus increasing the chance of a control being exposed to a case in this setting. Available risk factors for acquisition identified in previous studies were considered, together with novel risk factors for acquisition at the study institution (details in online supplementary material).

Independent predictors of KPCO acquisition were determined using multi-variate logistic regression with backwards selection (exit *P*>0.1), accounting for non-linear effects and interactions. For factors based on counts of events, the authors tested if the presence of any event, the total number of events or both were independently predictive. All analyses were conducted using Stata 14.1 (Stata Corp., College Station, TX, USA). Final model stability was assessed using bootstrapping (see online supplementary material for detailed statistical methods). As some cases may have been colonized or infected with KPCO at the time of transfer to the study institution but this was not detected within 48 h of admission (i.e. not true ‘acquisitions’), a sensitivity analysis was performed restricted to cases with a prior negative screen at the study institution.

### KPCO infection vs colonization

Risk factors for KPCO infection vs colonization among KPCO-positive cases were identified using a nested case–control study (Figure 1). Species were considered separately such that cases could contribute episodes of infection or colonization or both. KPCO infection episodes were defined by a KPCO clinical culture (non-perirectal surveillance) that met the National Healthcare Safety Network definition of clinical infection by chart review for pneumonia, bloodstream infection, urinary tract infection, intra-abdominal infection, and skin and soft tissue infection [16], and/or received antimicrobials targeting the site of infection by clinical culture. All other patients were considered to be colonized. The same potential predictors were considered as for acquisition, but excluding factors likely relevant to acquisition alone (patient location and KPCO colonization pressure), and also considering KPCO species. Exposures were calculated for the 90 days preceding the start of empiric treatment for infections, or to their last KPCO-positive culture for colonizations. Predictors of KPCO infection vs acquisition were determined using multi-variate logistic regression as above, allowing for within-patient correlation using robust standard errors.

### Fourteen-day mortality following KPCO infection

Information on vital status at 14 days post infection was available for all patients, including those with a first positive KPCO culture within 48 h of admission (i.e. likely imported cases) [17]. Predictors of 14-day all-cause mortality following each index infection (excluding repeat isolations within 14 days) were determined using Cox proportional hazards regression, allowing for within-patient correlation using robust standard errors. Given small numbers, no model selection was undertaken, and predictors were restricted *a priori* based on a review of the literature [[18], [19], [20]] (see online supplementary material for details).

### Ethics

This study was approved by the University of Virginia Health System with waiver of consent (IRB #18393, #18776 and #13558).

## Results

**Figure 2:**
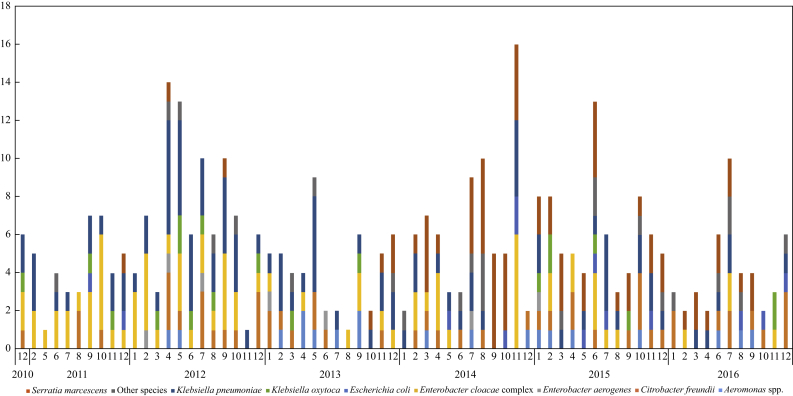
Incidence of unique patient and *Klebsiella pneumoniae* carbapenase-producing organisms over the study period. New colonizations/infections over time (unique species per patient). Note includes multiple infections/colonizations per patient when these are different species.

During the study, 43,748 perirectal screens for KPCO were undertaken at UVaHS in a total of 20,817 patients. Overall, 556 (1.3%) screens in 181 patients and 349 clinical samples in 151 additional patients were KPCO culture-positive. Twenty-nine patients were KPCO culture-positive at another institution or within 48 h of admission (i.e. likely acquired KPCO outside of UVaHS) and were excluded from acquisition analyses (Figure 1). In total, 303 patients acquired one or more KPCO species with a carbapenemase-positive phenotype >48 h post admission [274 confirmed by *bla*_KPC_ polymerase chain reaction (PCR); 29 discarded in error before performing PCR]. Sixty-one of the 303 (20%) cases had more than one species, with 368 distinct patient–KPCO species colonization/infections in total during the study period (Figure 2).

### Predictors of KPCO acquisition

The median age of cases was 59 [interquartile range (IQR) 49–69] years, and the median length of stay in the study institution was 19 (IQR 10–33) days in the 90 days prior to first KPCO isolation. The median age of controls was 62 (IQR 50–72) years, with a median of 12 (IQR 6–22) days of acute care hospital exposure at their last negative screen (*P*=0.06 and <0.001, respectively) (Table I).

**Table I:** Predictors of acquisition of *Klebsiella pneumoniae* carbapenemase-producing organisms (KPCO)

Variable	Controls (*N*=5929)		Cases (*N*=303)		Univariate			Multi-variate (all variables)			Final multi-variate model		
	*N*/median	%/IQR	*N*/median	%/IQR	Odds ratio	95% CI	*P*-value	Odds ratio	95% CI	*P*-value	Odds ratio	95% CI	*P*-value
Congestive heart failure	923	15.6%	49	16.2%	1.05	(0.76–1.43)	0.78	1.12	(0.74–1.68)	0.60			
Chronic lung disease	1140	19.2%	50	16.5%	0.83	(0.61–1.13)	0.24	0.88	(0.61–1.28)	0.50			
Liver disease	340	5.7%	25	8.3%	1.48	(0.97–2.26)	0.07	0.95	(0.47–1.93)	0.89			
Chronic kidney disease	1087	18.3%	70	23.1%	1.34	(1.02–1.76)	0.04	1.32	(0.87–2.00)	0.20			
Metastatic malignancy	306	5.2%	11	3.6%	0.69	(0.38–1.28)	0.24	0.93	(0.38–2.27)	0.88			
Human immunodeficiency virus	18	0.3%	1	0.3%	1.09	(0.14–8.17)	0.94	2.20	(0.27–17.99)	0.46			
Diabetes with complication	502	8.5%	32	10.6%	1.28	(0.88–1.86)	0.21	1.27	(0.75–2.14)	0.37			
Solid organ transplant	295	5.0%	24	7.9%	1.64	(1.07–2.53)	0.02	0.63	(0.26–1.48)	0.29			
Female	2636	44.5%	139	45.9%	1.06	(0.84–1.33)	0.63	1.21	(0.94–1.56)	0.14			
Department, vs other (reference)													
Other	3941	66.5%	163	53.8%	1.00			1.00					
STBICU	595	10.0%	60	19.8%	2.44	(1.79–3.32)	<0.001	1.26	(0.79–2.00)	0.34	1.19	(0.76–1.87)	0.45
MICU	405	6.8%	22	7.3%	1.31	(0.83–2.07)	0.24	0.64	(0.33–1.23)	0.18	0.61	(0.32–1.18)	0.14
LTACH	988	16.7%	58	19.1%	1.42	(1.04–1.93)	0.03	1.56	(0.62–3.95)	0.35	1.70	(0.69–4.21)	0.25
KPCO colonization pressure (STBICU)	0	0–0	0	0–0	1.04	(1.03–1.05)	<0.001	1.01	(0.99–1.03)	0.20	1.02	(1.00–1.03)	0.04
KPCO colonization pressure (MICU)	0	0–0	0	0–0	1.02	(1.00–1.04)	0.03	1.00	(0.97–1.02)	0.93	1.00	(0.98–1.03)	0.94
KPCO colonization pressure (LTACH)	0	0–0	0	0–0	1.00	(0.99–1.00)	0.22	1.00	(0.99–1.00)	0.32	1.00	(0.99–1.00)	0.26
KPCO colonization pressure (other unit)	2	0–10	0	0–6	0.99	(0.98–1.00)	0.06	0.99	(0.97–1.00)	0.06	0.99	(0.98–1.00)	0.06
Charlson score	1	0–4	1	0–4	1.01	(0.97–1.05)	0.73	0.96	(0.87–1.06)	0.40			
Age	62	50–72	59	49–69	0.99	(0.99–1.00)	0.06	1.00	(0.99–1.00)	0.31			
Acute hospital inpatient days	12	6–22	19	10–33	1.03	(1.03–1.04)	<0.001	1.00	(0.99–1.02)	0.71			
LTACH inpatient days	0	0–0	0	0–0									
(LTACH inpatient days)^−2^					0.86	(0.84–0.88)	<0.001	0.86	(0.84–0.89)	<0.001	0.87	(0.84–0.89)	<0.001
(LTACH inpatient days)^−1^					5.05	(3.78–6.75)	<0.001	5.20	(3.70–7.30)	<0.001	5.16	(3.71–7.18)	<0.001
Mechanical ventilation days	1	0–5	3	0–15	1.04	(1.03–1.05)	<0.001	1.03	(1.00–1.05)	0.02	1.02	(1.01–1.04)	0.005
Any aminoglycoside	242	4.1%	19	6.3%	1.57	(0.97–2.55)	0.07	0.95	(0.54–1.66)	0.86			
Any antifungal	1168	19.7%	115	38.0%	2.49	(1.96–3.17)	<0.001	1.01	(0.66–1.56)	0.95			
Antifungal days	0	0–0	0	0–6	1.09	(1.07–1.11)	<0.001	1.03	(0.99–1.08)	0.13			
Any beta-lactam/beta-lactamase inhibitor	1987	33.5%	152	50.2%	2.00	(1.58–2.52)	<0.001	1.68	(1.19–2.37)	0.003	1.69	(1.28–2.24)	<0.001
Beta-lactam/beta-lactamase inhibitor days	0	0–3	1	0–6	1.07	(1.04–1.09)	<0.001	0.96	(0.92–1.01)	0.10			
Any carbapenem	512	8.6%	63	20.8%	2.78	(2.07–3.72)	<0.001	1.47	(0.72–2.99)	0.29	2.56	(1.59–4.11)	<0.001
Carbapenem days	0	0–0	0	0–0	1.22	(1.15–1.29)	<0.001	0.99	(0.83–1.17)	0.91			
Any complex wound care	1854	31.3%	136	44.9%	1.79	(1.42–2.26)	<0.001	1.09	(0.78–1.52)	0.62			
Complex wound care days	0	0–1	0	0–2	1.27	(1.17–1.38)	<0.001	1.03	(0.87–1.23)	0.73			
Any complex abdominal pathology	409	6.9%	43	14.2%	2.23	(1.59–3.13)	<0.001	1.13	(0.74–1.72)	0.57			
Any complex thoracic pathology	455	7.7%	51	16.8%	2.43	(1.78–3.34)	<0.001	1.48	(1.01–2.15)	0.04	1.52	(1.06–2.19)	0.02
Any dialysis	740	12.5%	104	34.3%	3.66	(2.86–4.70)	<0.001	2.79	(1.81–4.29)	<0.001	2.96	(2.00–4.39)	<0.001
Dialysis days	0	0–0	0	0–2	1.11	(1.08–1.14)	<0.001	0.93	(0.87–0.99)	0.02	0.94	(0.89–1.00)	0.05
Any endoscopy	963	16.2%	82	27.1%	1.91	(1.47–2.49)	<0.001	1.24	(0.91–1.69)	0.18			
Any extended-spectrum cephalosporin	2633	44.4%	173	57.1%	1.67	(1.32–2.10)	<0.001	1.29	(0.93–1.78)	0.13			
Extended-spectrum cephalosporin days	0	0–5	2	0–7	1.04	(1.02–1.06)	<0.001	0.97	(0.94–1.01)	0.11			
Any fluroquinolone	1212	20.4%	79	26.1%	1.37	(1.05–1.79)	0.02	1.11	(0.73–1.69)	0.63			
Fluoroquinolone days	0	0–0	0	0–1	1.05	(1.00–1.10)	0.08	0.96	(0.88–1.06)	0.47			
Liver transplant	131	2.2%	17	5.6%	2.63	(1.57–4.42)	<0.001	2.13	(0.76–5.99)	0.15			
Kidney transplant	45	0.8%	2	0.7%	0.87	(0.21–3.60)	0.85	0.77	(0.16–3.80)	0.75			
Any transfusion	2859	48.2%	207	68.3%	2.32	(1.81–2.97)	<0.001	1.12	(0.81–1.57)	0.49			
Transfusion events	0	0–2	2	0–6	1.25	(1.20–1.30)	<0.001	1.07	(0.99–1.15)	0.11	1.09	(1.03–1.15)	0.002
Any enteral feeding	2792	47.1%	188	62.0%	1.84	(1.45–2.33)	<0.001	0.99	(0.71–1.37)	0.93			
Enteral feeding days	0	0–6	3	0–12	1.03	(1.02–1.04)	<0.001	0.99	(0.96–1.01)	0.23			
Any urinary catheter	948	16.0%	63	20.8%	1.38	(1.04–1.84)	0.03	1.16	(0.80–1.69)	0.43			
Urinary catheter days	0	0–0	0	0–0	1.22	(1.00–1.49)	0.04	1.36	(0.72–2.56)	0.34			
Any central vascular access	2708	45.7%	194	64.0%	2.12	(1.67–2.69)	<0.001	0.81	(0.58–1.14)	0.22			
Central vascular access events	0	0–1	1	0–3	1.57	(1.45–1.70)	<0.001	1.16	(0.97–1.38)	0.10			
Any beta-lactam/beta-lactamase inhibitor + any carbapenem (interaction *P*=0.006)											1.78	(1.09–2.89)	0.02

STBICU, surgical-trauma-burn intensive care unit; MICU, medical intensive care unit; LTACH, long-term acute care hospital; CI, confidence interval.

Note: see Supplementary Table 4 for bootstrap percentages and full multi-variate model. Location and cumulative days of KPCO colonization pressure by location forced into the model. (LTACH inpatient days)^−2^ and (LTACH inpatient days)^−1^ represent transformations of days of admission to the LTACH to allow a non-linear relationship (see Supplementary Figure S1).

Independent risk factors for KPCO acquisition (Table I) included mechanical ventilation [odds ratio (OR) per day=1.02, 95% confidence interval (CI) 1.01–1.04; *P*=0.005], use of carbapenems (OR=2.56, 95% CI 1.59–4.11; *P*<0.001) or β-lactamase/β-lactamase inhibitors (OR=1.69, 95% CI 1.28–2.24; *P*<0.001) or both (OR=1.78, 95% CI 1.09–2.89; *P*=0.02, *P*_interaction_=0.006), complex thoracic pathology (OR=1.52, 95% CI 1.06–2.19; *P*=0.02) and blood transfusions (OR per product received=1.09, 95% CI 1.03–1.15; *P*=0.002). Patients with any episode of dialysis had increased risk of acquisition (OR=2.96, 95% CI 2.00–4.39; *P*<0.001); however, risk decreased per additional dialysis episode received (OR per additional episode=0.94, 95% CI 0.89–1.00; *P*=0.05).

There was no independent effect of the unit where patients were tested (*P*=0.21). Risk of acquisition/infection increased per patient-day of KPCO colonization pressure on the STBICU (OR per patient-day=1.02, 95% CI 1.00–1.03; *P*=0.04) but not on the MICU (*P*=0.94) or LTACH (*P*=0.26) (*P*_interaction_=0.04). There was a trend towards KPCO colonization pressure being protective on other units, possibly reflecting the fact that controls in these locations were likely exposed to KPCO-positive patients by definition of the screening strategy around cases.

There was a non-linear relationship between time spent in the LTACH and risk of acquisition. Risk of acquisition was high immediately following LTACH admission, which frequently originated from the acute care hospital (i.e. acquisition was detected on LTACH admission screening), but then declined during the LTACH admission (Table I, Figure S1, see online supplementary material). After adjusting for all other predictors, there was no additional effect of the number of days spent in the acute care hospital (OR per day=1.00, 95% CI 0.99–1.02; *P*=0.67).

In addition to the variables included in the final model, extended-spectrum cephalosporin exposure, endoscopy, enteric feeding and vascular access events were included in ≥40% of bootstrap models used to assess model stability (Supplementary Table 4, see online supplementary material).

In the sensitivity analysis restricted to 208 cases with at least one prior negative screen (Supplementary Table 5, see online supplementary material), results were similar, with mechanical ventilation, β-lactamase/β-lactamase inhibitor, complicated thoracic pathology, dialysis, transfusions and LTACH-days included in the final model. As in the primary analysis, risk of acquisition increased per patient-day of exposure on the STBICU alone. Carbapenem exposure was not selected in the final model, but use of extended-spectrum cephalosporins and antifungals were selected instead.

### Predictors of KPCO infection vs colonization

Amongst the 303 cases, 368 distinct patient–KPCO species colonization/infections occurred (Table II). One hundred and twenty-two patients had a clinical culture, only 40 of whom had a KPCO-positive perirectal screen before their positive clinical culture, despite 87 (71%) having been screened in the prior 90 days. No typically environmental KPCO caused an infection, and therefore these species could not be included in the multi-variate analysis (*N*=21). Similarly, one patient with human immunodeficiency virus infection and one patient with a kidney transplant in the last 90 days predicted colonization and infection perfectly, respectively, and were therefore not included. Two patients had a novel Enterobacterales spp. identified and were also excluded from multi-variate analysis. Predictors of KPCO infection were assessed in the remaining 347 patient–KPCO episodes: 94 (27%) infections and 253 (73%) colonizations (Table III).

**Table II:** Species causing *Klebsiella pneumoniae* carbapenemase-producing organism (KPCO) infection vs colonization

	Total	Colonized		Infected	
		*N*	row %	*N*	row %
Included in analysis of risk factors for infection vs colonization					
*Klebsiella pneumoniae*	93	68	73%	25	27%
*Aeromonas* spp.	18	11	61%	7	39%
*Citrobacter freundii*	53	43	81%	10	19%
*Klebsiella aerogenes*	7	3	43%	4	57%
*Enterobacter cloacae* complex	76	53	70%	23	30%
*Escherichia coli*	14	11	79%	3	21%
*Klebsiella oxytoca*	19	14	74%	5	26%
*Serratia marcescens*	67	50	75%	17	25%
Colonization alone^a^					
*Citrobacter* spp. (non-*freundii*)	7	7	100%	0	0%
*Pantoea* spp.	5	5	100%	0	0%
*Raoultella* spp.	4	4	100%	0	0%
Other species^b^	5	5	100%	0	0%
Total	368	274		94	

a The breakdown of isolates which did not cause an infection and therefore excluded from the model as they predicted colonization perfectly.

b Other species were *Kluyvera intermedia* (*N*=1), *Morganella morganii* (*N*=1), *Proteus mirabilis* (*N*=1) and unknown species of Enterobacterales (*N*=2) which could not be speciated further in a clinical laboratory.

**Table III:** Predictors of *Klebsiella pneumoniae* carbapenemase-producing organism (KPCO) infection vs colonization including multiple species

Variable	Colonized (*N*=253)		Infected (*N*=94)		Univariate			Multi-variate (all variables)			Final multi-variate model		
	*N*/median	%/IQR	*N*/median	%/IQR	Odds ratio	95% CI	*P*-value	Odds ratio	95% CI	*P*-value	Odds ratio	95% CI	*P*-value
Congestive heart failure	49	19%	11	12%	0.55	(0.29–1.07)	0.08	0.74	(0.30–1.84)	0.52			
Chronic lung disease	53	21%	11	12%	0.50	(0.25–0.99)	0.05	0.69	(0.28–1.72)	0.42			
Chronic kidney disease	64	25%	31	33%	1.45	(0.89–2.38)	0.14	2.06	(0.79–5.34)	0.14			
Metastatic malignancy	6	2%	7	7%	3.31	(1.29–8.48)	0.01	5.75	(0.85–38.95)	0.07	4.26	(1.27–14.25)	0.02
Diabetes with complication	35	14%	10	11%	0.74	(0.37–1.48)	0.39	0.98	(0.27–3.54)	0.98			
Solid organ transplant	19	8%	15	16%	2.34	(1.15–4.74)	0.02	3.23	(0.94–11.12)	0.06			
Female	119	47%	46	49%	1.08	(0.68–1.72)	0.75	1.47	(0.76–2.83)	0.25	1.60	(0.96–2.69)	0.07
Species, vs *Klebsiella pneumoniae* (reference)^a^													
*Klebsiella pneumoniae*	68	27%	25	27%	1.00			1.00					
*Aeromonas* spp.	11	4%	7	7%	1.73	(0.61–4.90)	0.30	1.37	(0.46–4.03)	0.57			
*Citrobacter freundii*	43	17%	10	11%	0.63	(0.28–1.44)	0.28	0.58	(0.20–1.70)	0.32			
*Klebsiella aerogenes*	3	1%	4	4%	3.63	(0.74–17.72)	0.11	3.78	(0.58–24.52)	0.16			
*Enterobacter cloacae* complex	53	21%	23	24%	1.18	(0.61–2.30)	0.63	1.62	(0.73–3.58)	0.23			
*Escherichia coli*	11	4%	3	3%	0.74	(0.19–2.87)	0.67	0.63	(0.12–3.30)	0.59			
*Klebsiella oxytoca*	14	6%	5	5%	0.97	(0.31–3.03)	0.96	1.13	(0.23–5.65)	0.88			
*Serratia marcescens*	50	20%	17	18%	0.92	(0.45–1.90)	0.83	0.45	(0.17–1.18)	0.10			
Charlson score	2	0–4	2	0–4	1.03	(0.96–1.12)	0.40	0.97	(0.78–1.20)	0.76			
Age	58	49–68	58.5	45–68	0.99	(0.98–1.01)	0.40	0.99	(0.97–1.01)	0.40			
Acute inpatient days	20	11–33	28	14–46	1.02	(1.01–1.03)	<0.001	1.02	(0.99–1.05)	0.20	1.02	(1.00–1.04)	0.04
LTACH inpatient days	0	0–0	0	0–0	0.99	(0.97–1.01)	0.34	1.00	(0.97–1.04)	0.82			
Any aminoglycoside	21	8%	13	14%	1.77	(0.94–3.35)	0.08	2.13	(0.52–8.69)	0.29			
Aminoglycoside days	0	0–0	0	0–0	1.24	(0.97–1.59)	0.08	0.65	(0.24–1.74)	0.39			
Any antifungal	103	41%	49	52%	1.59	(1.00–2.53)	0.05	0.82	(0.29–2.34)	0.71			
Antifungal days	0	0–7	2	0–17	1.03	(1.01–1.05)	0.003	1.00	(0.94–1.06)	0.97			
Any beta-lactam/beta-lactamase inhibitor	131	52%	50	53%	1.06	(0.66–1.69)	0.81	0.92	(0.43–1.97)	0.83			
Beta-lactam/beta-lactamase inhibitor days	1	0–7	1	0–6	0.99	(0.95–1.02)	0.46	0.94	(0.88–1.01)	0.08	0.94	(0.90–0.98)	0.004
Any carbapenem	56	22%	32	34%	1.82	(1.09–3.01)	0.02	1.10	(0.33–3.64)	0.87			
Carbapenem days	0	0–0	0	0–7	1.09	(1.04–1.14)	<0.001	1.03	(0.90–1.17)	0.69			
Any complex wound care	120	47%	55	59%	1.56	(0.98–2.50)	0.06	1.11	(0.52–2.38)	0.78			
Complex wound care days	0	0–2	1	0–3	1.15	(1.03–1.29)	0.02	1.16	(0.90–1.48)	0.25			
Any complex abdominal pathology	29	11%	26	28%	2.95	(1.68–5.18)	<0.001	2.19	(0.91–5.24)	0.08	2.51	(1.26–4.99)	0.009
Any complex thoracic pathology	41	16%	19	20%	1.31	(0.72–2.38)	0.38	0.52	(0.16–1.66)	0.27			
Complex thoracic pathology days	0	0–0	0	0–0	1.24	(0.84–1.85)	0.28	1.53	(0.30–7.73)	0.60			
Any dialysis	83	33%	52	55%	2.54	(1.59–4.05)	<0.001	2.97	(1.16–7.58)	0.02	2.77	(1.37–5.60)	0.004
Dialysis days	0	0–3	1	0–4	1.02	(0.99–1.06)	0.14	0.91	(0.85–0.98)	0.007	0.94	(0.90–0.98)	0.003
Any endoscopy	68	27%	31	33%	1.34	(0.82–2.19)	0.24	0.53	(0.23–1.22)	0.14			
Endoscopy events	0	0–1	0	0–1	1.26	(0.99–1.60)	0.06	1.50	(0.78–2.86)	0.22			
Any extended-spectrum cephalosporin	141	56%	63	67%	1.61	(0.99–2.63)	0.05	1.02	(0.48–2.18)	0.95			
Extended-spectrum cephalosporin days	1	0–7	3	0–17	1.04	(1.01–1.07)	0.003	1.00	(0.94–1.07)	0.98			
Any fluroquinolone	64	25%	39	41%	2.09	(1.29–3.41)	0.003	1.13	(0.53–2.42)	0.76			
Fluoroquinolone days	0	0–1	0	0–3	1.10	(1.03–1.17)	0.002	1.02	(0.90–1.14)	0.77			
Any enteral feeding	156	62%	64	68%	1.33	(0.82–2.15)	0.25	1.02	(0.43–2.46)	0.96			
Enteral feeding days	3	0–15	5	0–22	1.01	(1.00–1.03)	0.19	0.97	(0.94–1.01)	0.12			
Any urinary catheter	58	23%	24	26%	1.15	(0.67–1.98)	0.61	1.85	(0.90–3.83)	0.10			
Urinary catheter days	0	0–0	0	0–1	0.99	(0.75–1.32)	0.96	0.67	(0.27–1.68)	0.39			
Any central vascular access	174	69%	76	81%	1.92	(1.09–3.38)	0.02	1.17	(0.49–2.81)	0.73			
Central vascular access events	1	0–3	2	1–4	1.19	(1.09–1.30)	<0.001	0.99	(0.80–1.22)	0.92			
Any mechanical ventilation	160	63%	69	73%	1.60	(0.96–2.67)	0.07	1.44	(0.59–3.56)	0.42			
Mechanical ventilation days	3	0–13	10	0–24	1.03	(1.01–1.04)	0.001	1.02	(0.98–1.06)	0.42			
Any transfusion	184	73%	71	76%	1.16	(0.68–1.98)	0.59	0.29	(0.13–0.64)	0.002	0.41	(0.20–0.80)	0.004
Transfusion events	2	0–5	4.5	1–13	1.09	(1.05–1.13)	<0.001	1.09	(0.99–1.21)	0.07	1.09	(1.02–1.16)	0.003

LTACH, long-term acute care hospital; CI, confidence interval.

Note: excluding isolates from other species, patients with human immunodeficiency virus and renal transplant patients as these predicted infection/colonization perfectly.

a Some patients may have more than one isolate across species. The breakdown of isolates which caused an infection. Species which did not cause an infection were excluded as they perfectly predicted colonization alone.

Independent predictors of KPCO infection (Table III) were metastatic malignancy (OR=4.26, 95% CI 1.27–14.25; *P*=0.02), longer acute care hospital inpatient stay (OR per day=1.02, 95% CI 1.001–1.04; *P*=0.04), and complex intra-abdominal pathology (OR=2.51, 95% CI 1.26–4.99; *P*=0.009). Ever having dialysis was associated with increased risk of infection (OR=2.77, 95% CI 1.37–5.60; *P*=0.004), but this declined per additional day of dialysis received (OR per additional day=0.94, 95% CI 0.90–0.98; *P*=0.003). Risk of infection was higher among those who had never received blood product transfusion (OR=2.47, 95% CI 1.25–4.89; *P*=0.01), but increased per transfusion event (OR per event=1.09, 95% CI 1.02–1.16; P=0.01). β-lactam/β-lactamase inhibitor exposure decreased the risk of infection vs colonization (OR=0.94 per additional day, 95% CI 0.90–0.98; *P*=0.004).

After adjusting for these predictors, there was no evidence of an additional effect of the most common bacterial species [overall *P*=0.21, compared with *K. pneumoniae*, OR (95% CI, *P*-value), *Aeromonas* spp. 1.22 (0.37–4.02; *P*=0.74), *Citrobacter freundii* 0.53 (0.21–1.32; *P*=0.17), *E. aerogenes* 2.69 (0.50–14.6; *P*=0.25), *E. cloacae* complex 1.37 (0.65–2.89; *P*=0.41), *E. coli* 0.66 (0.15–2.90; *P*=0.59), *K. oxytoca* 1.27 (0.36–4.47; *P*=0.71) and *S. marcescens* 0.49 (0.21–1.14; *P*=0.10)]. *Raoutella* spp., *Pantoea* spp. and *Citrobacter* non-*freundii* complex perfectly predicted colonization alone (Table II). There was no evidence of additional effects of days of carbapenem, extended-spectrum cephalosporin or fluoroquinolone exposure (*P*=0.56, 0.21 and 0.19, respectively).

### Predictors of 14-day mortality following KPCO infection

Including patients admitted with KPCO acquisition that plausibly occurred elsewhere (i.e. identified during the first 48 h of their first hospital stay or before admission), 143 KPCO infections were identified in 100 patients. Of these, 46 (32%) were urinary tract infections (all surviving 14 days post infection), 50 (35%) were intra-abdominal infections, 25 (17%) were pneumonia, 20 (14%) were bacteraemia, one (<1%) was tracheobronchitis and one (<1%) following a skin and soft tissue infection. In 97 non-urinary infections, 20 (21%) patients died within 14 days (10 following intra-abdominal infection, seven following pneumonia, two following bacteraemia, and one with a skin and soft tissue infection). On multi-variate analysis, excluding patients with a urinary tract infection (none of whom died) (Table IV), source control was associated with reduced 14-day mortality risk [hazard ratio (HR)=0.07, 95% CI 0.01–0.44; *P*=0.005] and there was a trend towards lower mortality with active therapy (HR=0.32, 95% CI 0.09–1.10; *P*=0.07). There was also a very weak association between infection with an intrinsically colistin-resistant KPCO (i.e. *S. marcescens*) and mortality independent of active therapy (HR=1.97, 95% CI 0.83–4.68; *P*=0.12).

**Table IV:** Predictors of 14-day mortality following infection with *Klebsiella pneumoniae* carbapenemase-producing organisms (KPCO)

Variable	Alive at 14 days (*N*=77)		Died by 14 days (*N*=20)		Univariate			Multi-variate		
	*N*/median	%/IQR	*N*/median	%/IQR	Hazard ratio	95% CI	*P*-value	Hazard ratio	95% CI	*P*-value
Age, per year	57	46–65	57	43–66	0.99	(0.96–1.03)	0.64	1.00	(0.97–1.03)	0.84
Female	29	38%	4	20%	0.44	(0.16–1.21)	0.11	0.47	(0.15–1.42)	0.18
Active therapy	67	87%	11	55%	0.23	(0.08–0.62)	0.004	0.32	(0.09–1.10)	0.07
Source control	43	56%	1	5%	0.05	(0.01–0.39)	0.004	0.07	(0.01–0.44)	0.005
Number of previous KPCO infections, per infection	0	0–1	0	0–1	0.99	(0.64–1.53)	0.96	0.76	(0.47–1.24)	0.27
Infection with intrinsically colistin-resistant KPCO	13	17%	9	45%	3.05	(1.39–6.69)	0.005	1.97	(0.83–4.68)	0.12
Charlson score, per point increase	2	0–5	4	0.5–6	1.10	(0.98–1.24)	0.10	0.98	(0.84–1.15)	0.81
Infection focus, intra-abdominal (baseline)	#N/A	#N/A	#N/A	#N/A	1.00			1.00		
Bacteraemia	18	23%	2	10%	0.48	(0.10–2.22)	0.35	1.12	(0.30–4.21)	0.86
Pneumonia	18	23%	7	35%	2.37	(0.49–11.47)	0.29	0.74	(0.23–2.43)	0.62
Other	1	1%	1	5%	1.54	(0.55–4.34)	0.41	2.12	(0.54–8.30)	0.28

CI, confidence interval.

Note: not including 46 patients with KPCO urinary tract infections, none of whom died within 14 days.

## Discussion

To the authors' knowledge, this is the largest study to date to examine clinical risk factors for acquisition of, infection with and mortality following multi-species KPCO in a single institution over several years under endemic conditions with a robust perirectal screening programme. This allowed the quantification of risks in the context of multi-species KPCO transmission (i.e. focusing on resistance genes as opposed to resistant strains), which is becoming increasingly common [21,22]. The findings are therefore relevant to CPE outbreak management guidelines and stratifying patients for screening and treatment, especially when hospital environment may play a role [15,23,24].

One important finding is the variable risk for acquisition associated with exposure to other colonized/infected patients (i.e. a proxy marker for transmission between patients). Exposure to other KPCO-colonized/-infected patients increased the risk of acquisition in the STBICU but not elsewhere in the hospital, supporting a role of other sources. Five studies have found that exposure to another CPE-colonized patient increases the risk of acquisition; however, all were in KPC-producing *K. pneumoniae* outbreaks [7,8,[25], [26], [27], [28]]. In the multi-species KPCO setting of the present study, additional multi-factorial modes of acquisition and other unsampled reservoir(s) could include: missed, silently colonized patients [either the wrong patients were screened and/or laboratory methods lack sensitivity (the method used has reported microbiological sensitivity of 85.7%)] [29]; colonized staff; or other environmental reservoirs varying by unit. Environmental wastewater reservoirs have almost certainly played a role in endemic transmission in the study institution [15], as elsewhere [12,13,23,24]. Similar findings have been noted in transmission studies of extended-spectrum β-lactamase (ESBL)-producing Enterobacterales, where interventions to prevent patient-to-patient transmission have been ineffective in preventing acquisition [30]. The present findings indicate that polyclonal/multi-species CPE outbreaks may require novel screening and isolation approaches paired with environmental interventions [6]. Furthermore, the results call into question the potential efficacy of some interventions, such as patient and staff cohorting, advocated in clonal, single-species outbreaks where patient-to-patient transmission likely plays a predominant role [6]. The present study also found that KPCO acquisition was limited within the LTACH; most detected acquisition occurred shortly after admission, suggesting importation from the acute care hospital. This highlights that acquisitions from other KPCO-positive patients can be minimized. This may be due to the aggressive infection control measures in place at the LTACH with all patients on contact precautions and weekly CPE surveillance, as described above [31].

Given the role of horizontal gene transfer in CPE dissemination, no attempt was made to perform species or genomic linkage between patients in this study, but a previous, large genomic analysis of the outbreak suggested that patient-to-patient transmission of genetically-related strains accounted for only a minority (48/167; 29%) of transmission events [11].

Apart from KPCO colonization pressure, this study confirms that risk factors for multi-species KPCO acquisition generally mirror those identified from clonal carbapenemase-producing *K. pneumoniae* outbreaks. Acquisition in both contexts occurs in vulnerable patients who are critically ill and exposed to broad-spectrum antibiotics [7,8,25,32]. Several novel risk factors associated with KPCO acquisition were also identified in the study setting, namely transfusion, dialysis and complex thoracic pathology. Short-term dialysis was associated with greatest risk, reflecting these patients may be critically ill and dialyse through temporary vascular access. Additionally, temporary dialysis was performed in the room of a critically ill patient with effluent draining continually into wastewater, possibly increasing nutrient exposure and bacterial loads in the wastewater. Transfusion and complex thoracic pathology may also be markers for complications in surgical patients with multiple interventions. The thoracic procedures were related to empyema, need for chest tube and decortication procedures, and procedures to control haemorrhage or infection from an initial surgery which often occurred in patients with complications.

Despite the extensive perirectal screening programme conducting over 6500 screens per year [29], only a minority of patients (33%) with a KPCO-positive clinical culture had previously been identified as KPCO-colonized. However, the majority (71%) had had an antecedent perirectal screen, suggesting that the screening strategy is targeting the correct population. Screening may not be sufficiently frequent, or culture may be insufficiently sensitive. The analyses of KPCO infection risk focused on comparing those who were colonized without experiencing invasion with those who developed invasive infection. Findings are therefore most generalizable to KPCO-colonized patients who may develop invasive infection rather than *a priori* general hospital populations developing invasive infection with KPCO vs other pathogens. However, as colonization (even if short-lived) is generally assumed to precede invasion, and, as above, prior colonization may have been missed due to relatively infrequent screening, the study approach is more efficient for identifying factors genuinely associated with KPCO invasion. As in previous studies, infection rather than colonization was more likely in patients with multiple co-morbidities, including prolonged hospitalization, metastatic malignancy, and complicated intra-abdominal pathology, often with multiple surgical revisions [[33], [34], [35]]. Unlike other studies, however, increasing antibiotic exposure was not a risk factor for infection, which may represent high antimicrobial exposure in the high-risk control group [36]. Infection was unsurprisingly associated with pathogenic species; however, colonization with less pathogenic organisms such as *Raoutella* spp. or *Kluyvera* spp. may have an important role to play in resistance gene transfer to more pathogenic organisms, environmental persistence and transmission, and is not typically detected under current screening guidelines [6]. These data may help guide clinicians to determine, in colonized or very-high-risk patients, which patients are most likely to develop invasive infection, and thus those who might benefit from including KPCO active agents in an empiric treatment regimen.

Source control was the only significant predictor of 14-day mortality and is an important, potentially modifiable risk [18]. None of 46 episodes of urinary tract infection led to death within 14 days, suggesting that this represents a lower risk clinical category, supported by other comparisons of carbapenem-resistant *K. pneumoniae* [37]. With relatively small numbers of deaths, power was low to detect effects of other factors, but active therapy tended to be associated with lower mortality risk, and *S. marcescens* infection, which carries intrinsic colistin resistance, tended to be associated with greater risk, as in other studies [38].

This study has several limitations. Firstly, it is a retrospective study of a single medical system, and may not be generalizable in all respects to other centres. Small numbers, particularly for the mortality analysis, likely limited power to detect relevant risk factors. Genetic analyses of strains might refine the assessment of relevant KPCO colonization pressures (although the complexities posed by horizontal gene transfer would need to be addressed), as would detailed contemporaneous sampling of other reservoirs (e.g. environment, staff). Finally, given the complex nature of medical records and the patient group being surveyed, classification of procedures into distinct subcategories that could be assessed in regression models was not straightforward.

In conclusion, to the authors' knowledge, this is the largest study to date of acquisition, infection and mortality risks associated with multi-species CPE in a single centre. The study demonstrated overlapping and unique risk factors associated with acquisition of multiple species of KPCO compared with prior evaluations which focused on single clones/species (often *K. pneumoniae*). A particularly important finding was that risk of acquisition was not universally associated with exposure to other KPCO-colonized patients [7,27,28], and that CPE management guidelines may need to be more nuanced for multi-species CPE transmission linked by the same resistance gene. Future work to investigate the role of non-patient reservoirs in the hospital environment which can act as a source of these organisms is essential [12].

## Conflict of interest statement

None declared.

## Funding sources

This work was funded, in part, by a contract from the Centres for Disease Control and Prevention Broad Agency Announcement BAA 2016-N-17812. ASW, DWC, TEAP and ASW are affiliated to the 10.13039/501100012091National Institute for Health Research (NIHR) Health Protection Research Unit in Healthcare Associated Infections and Antimicrobial Resistance at the 10.13039/501100000718University of Oxford in partnership with 10.13039/501100002141Public Health England (Grant HPRU-2012-10041) and are supported by the Oxford NIHR Biomedical Research Centre. ASW and TEAP are NIHR Senior Investigators. The views expressed are those of the authors and not necessarily those of the National Health Service, the NIHR, the Department of Health or PHE. DWE is a Robertson Foundation Big Data Fellow.
