# Supplementary material for: Risk factors for Klebsiella pneumoniae carbapenemase (KPC) gene acquisition and clinical outcomes across multiple bacterial species
Source: J Hosp Infect. 2020 Apr;104(4):456–68. doi: 10.1016/j.jhin.2020.01.005 (PMC7193892; doi:10.1016/j.jhin.2020.01.005)
Supplement: Supplementary file 1 [file mmc1.docx]

# **Supplement**

## **Supplementary Methods**

### **KPCO colonisation pressure**

KPCO-colonisation pressure was calculated irrespective of species (i.e. effectively as a marker of KPC resistance gene pressure) as the sum of the total number of patient-days spent by other KPCO colonised and infected patients on the same unit as the case/control over the preceding 90 days (e.g. three KPCO-colonised patients on the same unit on one day and two on the next was five patient-day exposures).

### **KPCO acquisition**

Independent predictors of KPCO acquisition were determined using multivariate logistic regression with backwards selection (exit p>0.1). Variables with p<0.1 were included to control for confounding, with only factors with p<0.05 reported. Patient location at testing, and cumulative patient-days of KPCO-colonisation pressure by location at testing (interaction), were forced into models to account for the differential screening strategies (described above). Fractional polynomials were used to incorporate non-linear effects (Stata mfp), truncating continuous variables at the 95^th^ percentile. Following backwards elimination, each excluded variable was added back to the model and retained if p<0.1. Pairwise interactions were then investigated and retained if p<0.01. All analyses were conducted using Stata 14.1 (Stata Corp., College Station, TX). Final model stability was assessed using bootstrap (Stata mfpboot) (n=200). (See supplement for further details.)

### **14-day mortality following KPCO infection**

Given small numbers, no model selection was undertaken, and predictors were restricted *a priori* based on literature review[18-20] to age, sex, Charlson comorbidity index, an infecting species with intrinsic colistin resistance (e.g. *Serratia marcescens*), number of previous KPCO infections, receipt of active antimicrobials, and source control (defined as line removal for central line bloodstream infections and percutaneous/surgical drainage of any infected fluid collection)[17, 18]. Active therapy was defined following the Clinical Laboratory and Standards Institute (non-tigecycline) or Food and Drug Administration (tigecycline) criteria[21].

### **Laboratory and screening procedures**

Over the study period weekly peri-rectal sweeps were performed on all patients admitted to the long-term acute care hospital (LTACH), the surgical (SICU) and medical (MICU) intensive care units as well as any unit where another patient who was known to be colonised or infected was admitted using methods previously described [1]. From December 1^st^, 2010 to October 15^th^, 2014, isolates underwent KPC polymerase chain reaction (PCR) as previously described [2]. Isolates underwent KPC PCR confirmation using BDMax (Becton Dickinson, Franklin Lakes, NJ) from October 16^th^, 2014 until July 2016 and then CarbaR (Cepheid Sunnyvale, CA) from July 2016 until Jan 2017. Positive and negative quality controls were run weekly according to the manufacturers’ instructions and other carbapenemase enzymes were excluded from the study (i.e. did not include two patients with *bla*_OXA-48-like_ *K. pneumoniae*[3] and otherwise did not see other unique carbapenemase genes).

All speciation was performed using a combination of VITEK2, VITEK-MS (Biomerieux, Durham, NC), and routine biochemical tests. Susceptibility testing was done by various methods over the study period which included disk diffusion and VITEK2-AST-GN70 cards. Tigecycline susceptibility was assessed via disk diffusion until April 2012 and then VITEK2 thereafter according to the Clinical Laboratory and Standards Document [4] or manufacturer recommendations. Colistin susceptibility was only tested by disk diffusion without interpretation. Ceftazidime-avibactam was tested after May, 2016 by broth microdilution at Lab Specialist (Dayton, Ohio). Where interpretive criteria were applied the Clinical Laboratory and Standards Document m100 was used [5] except for tigecycline where FDA criteria were used.

### **Identification of previous and novel risk factors for acquisition**

A survey of the literature describing risk factors for KPCO acquisition is summarised in Supplementary Table 1. Where applicable, each risk factor was mapped through expert guidance to specific entries contained in a single clinical or administrative system in an attempt to avoid multiple representations of the same data (see Supplementary Table 2 for a description of the data sources). Mobility, diaper use, other infections, and other multidrug resistant organisms were excluded due to inability to find accurate representations in any of the electronic systems. Included factors based on medication administration records may represent the risk which had been previously described [6] more accurately in our study.

Novel risk factors to be included in multivariate analyses with those in Table 1 were then identified from all the medication and invasive procedures codes contained in the clinical systems. As this was a preliminary analysis, a less restrictive definition of controls (all patients with a single negative peri-rectal screen who remained negative throughout the study period rather than enforcing two screens in the same hospital stay) was used to screen novel codes. As in the main analysis, patients with first KPCO isolation within 48 hours of their first stay within University of Virginia Health System (UVaHS) were identified as imports and excluded. All other patients with KPCO isolations were labeled as cases (acquisitions) and factors were derived from a 90 day look back period. Once identified, a random 70% of cases and controls with first sample collection dates between December 1, 2010 and March 6, 2015 were selected to allow for out of sample validation. Procedures and medications in the 90 days prior to the first sample collection date were calculated, and the prevalence of each procedure or medication associated with 5 or more cases compared between cases and controls using Fisher’s exact tests, using a Holm-Bonferroni adjustment to resulting p values to account for multiple comparison, and a 0.05 adjusted cutoff to assess significance. Clinical expert guidance was then used to either exclude procedures based on clinical implausibility as a risk factor (e.g. certain laboratory tests were significant but unlikely to be a source of acquisition and thus considered to represent another co-morbidity and were therefore excluded) or to group individual procedures/medications, to which further review mapped additional relevant procedures (see below). This identified the following groups subsequently considered in the main case-control study (together with other risk factors from the literature): complex wound care, antifungals, complicated cardiothoracic pathology, dialysis, transfusion of blood products, tube-feed related procedures.

Sensitivity analyses considered look back windows of 30 and 180 days. Results were very similar between 90 days and 180 days, whereas similar predictors had smaller impacts using 30 days suggesting that this shorter window was not capturing the risk associated with prolonged complicated admissions (data not shown).

Supplementary Figure S1: Non-linear relationship between days of admission to the Long Term Acute Care Hospital (LTACH) and risk of colonisation.


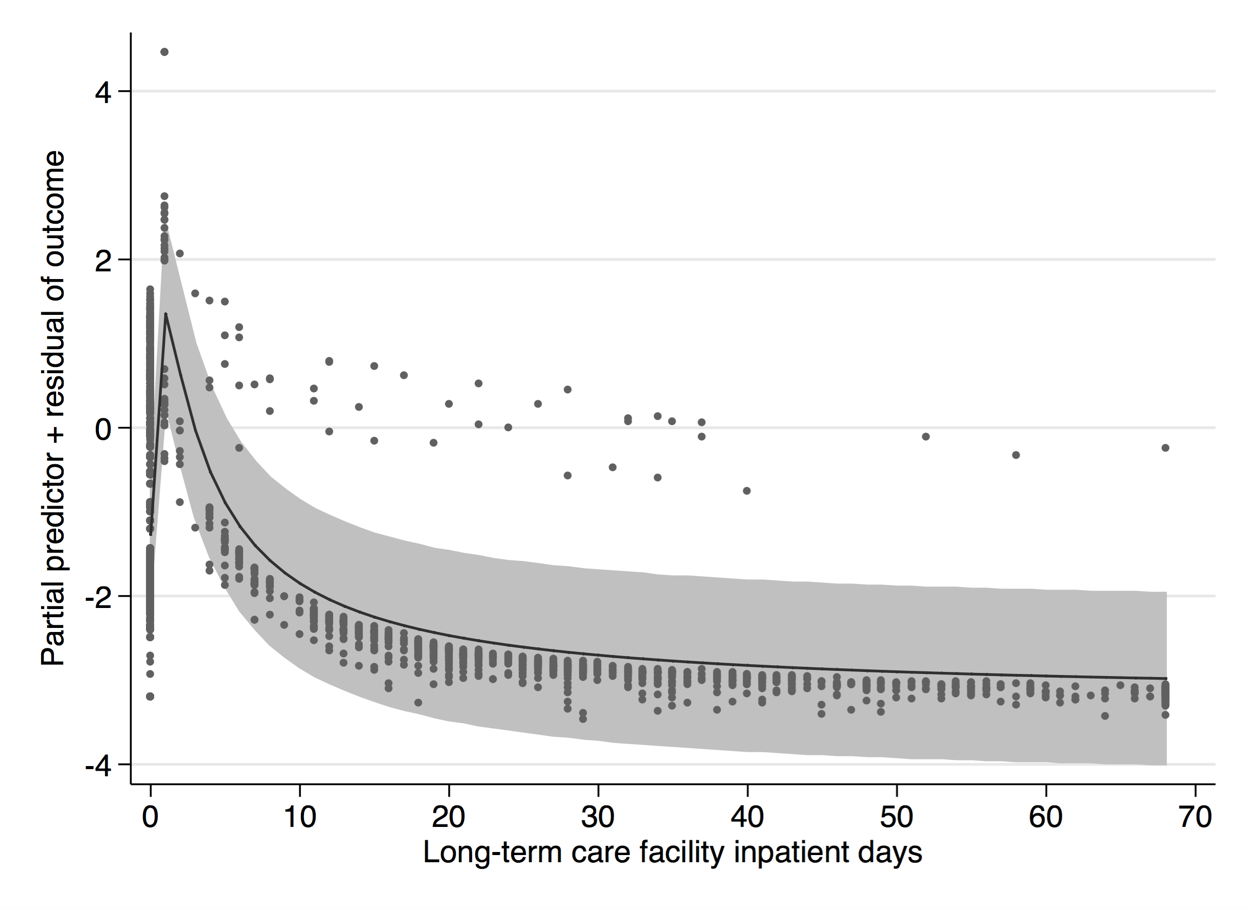


Supplementary Table 1: Risk factors for acquisition of *Klebsiella pneumoniae* carbapenemase-producing organisms identified from literature

| Factor in current analysis | Factor from the literature | Source | Included in this analysis | Data source |
| --- | --- | --- | --- | --- |
| **Complicated abdominal pathology** | Complicated abdominal pathology | [7] | Yes | Epic Clinical Procedures |
| **Endoscopy** | Endoscope or bronchoscopy exposure | [8] | Yes | Hyperion CPT® |
| **Mechanical ventilation** | Tracheostomy | [9], [10], [11], [12] | Yes | Hyperion CPT® |
| **Mechanical ventilation** | Mechanical ventilation | [13],[14] [15], [16, 17] | Yes | Hyperion CPT® |
| **Vascular access** | Central vascular access | [9], [16],[18],[19], [20], [12], [21], [22] | Yes | Hyperion CPT® |
| **Urinary catheter** | Urinary catheter | [17, 18],[19], [14], [20], [12] | Yes | Hyperion CPT® and HCPCS ICD-9 |
| **Liver transplant** | Liver transplant | [16], [7] | Yes | Hyperion Billing Systems |
| **Kidney transplant** | Kidney transplant | [16] | Yes | Hyperion Billing Systems |
| **Separate drug classification:**  **aminoglycosides,**  **antifungals,**  **β-lactam / β-lactamase Inhibitors (BL-BLI),**  **carbapenems,**  **extended spectrum intravenous β-lactams (ESIBL),**  **fluoroquinolones** | Anti-Infective | [23], [9], [10], [14], [15], [24], [25], [6], [26], [16], [17], [18, 27], [28], [19], [11], [29], | Yes | Epic Medical Administration Records |
| **Charlson score, (premorbid conditions)** | Charlson Score | [13], [15], [16], [18], [19] | Yes | Hyperion HCPCS ICD9/10 |
| **Location:**  **surgical ICU (SICU), medical ICU (MICU), long term (LTACH), others (OTHER)** | Intensive care unit stay/severe illness, long tern acute care hospital | [23], [26], [6], [16],[17], [27], [20], [11], [29] | Yes | Siemens INVISION® |
| **Number of inpatient days in last 90 days** | Prolonged length of stay in hospital | [23], [24], [16], [30], [14, 19] | Yes | Siemens INVISION® |
| **COPD** | Chronic pulmonary disease | [23], [15] | Yes | Hyperion HCPCS - ICD9/10 |
| **Diabetes mellitus** | Diabetes mellitus with complications | [17] | Yes | Hyperion HCPCS - ICD9/10 |
| **Malignancy** | Active malignancy | [27] | Yes | Hyperion HCPCS - ICD9/10 |
| **Female** | Male gender | [9] | Yes | Epic Clarity |
| **Age** | Age | [28], [22] | Yes | Epic Clarity |
| **Colonisation pressure from other patients with CRE** | Number of days of exposure to another patient with KPCO* | [9], [13], [15], [24], [27] | Yes | Siemens INVISION® |
| **Any KPCO screen before prior positive** | Screening within 90 days of the first culture growing KPCO | [23], [9], [10], [15], [24], [25] [6] | No because included in definition of “control” | Sunquest Information Systems |
| **N/A** | Mobility | [13] | No | Not accurately represented in the electronic systems |
| **N/A** | Diaper use | [24] | No | Not accurately represented in electronic systems |
| **N/A** | Other infection | [6, 17], [27] | No | Not accurately represented in the electronic systems |
| **N/A** | Other multidrug resistant organism | [13] | No | Not accurately represented in the electronic systems |
| **N/A** | Admission for another country | [30] | No | Not accurately represented in the electronic systems |
| **N/A** | Having offspring | [22] | No | Not accurately represented in the electronic systems |

### **Data sources**

Once the set of categories was determined through surveys of literature and analysis of procedures/medications described above, related procedures and medications were mapped to them from the clinical and administrative systems. Epic groupers were used for medications and mixtures. Related clinical procedures were found through expert guidance and chart review. Administrative procedure codes used standard groupings of Current Procedural Terminology (CPT®) codes, Healthcare Common Procedure Coding System (HCPCS) codes, and International Classification of Diseases (ICD-9 and ICD-10) diagnosis and procedure codes where possible. A list of clinical and administrative systems used in this study is presented in Supplementary Table 2, and the entire mapping of each category to the individual clinical medications, clinical procedures, and administrative procedures is presented in Supplementary Table 3.

The risk factors are derived from data originating from multiple sources: the Epic™ hospital electronic medical record (EMR) system, Siemans INVISION®, and Hyperion Billing Systems codes (CPT® and HCPCS). Accounting system records provide most of the procedure data in the form of CPT® and HCPC codes. The clinical system is used to accurately describe medication administration, and to provide procedures for some of the factors that do not appear in the accounting system. For example, we found that laparotomy procedures were effectively captured within clinical data but were missing from administrative codes due to being one small part of a many different surgical procedures. Using a combination of these two systems, 17 (6%) of the cases and 478 (8%) of the controls used for feature selection had none of the medication or procedure risk factors during the preceding 90 days.

Supplementary Table 2: Data sources used within the study

| Source Description | Source |
| --- | --- |
| Epic™ Clarity Clinical Medical Administration Record | MAR |
| GE Centricity™ Surgical Procedures | Operating Room Procedure |
| Epic™ Clarity Clinical Procedures | Procedure |
| Hyperion Billing Solutions CPT® Codes | CPT |
| Hyperion Billing Solutions HCPCS Codes | ICD9PCDR |
| Siemans INVISION® / A2K3 bed tracking | Siemans INVISION® |

Supplementary Table 3: Mapping of codes to higher level categories used within the study

| Category | Code / Description | Source |
| --- | --- | --- |
| Aminoglycoside | AMIKACIN | MAR |
| Aminoglycoside | GENTAMICIN | MAR |
| Aminoglycoside | TOBRAMYCIN | MAR |
| Antifungal | AMPHOTERICIN B | MAR |
| Antifungal | AMPHOTRICIN B LIPOSOMAL | MAR |
| Antifungal | ANIDULAFUNGIN | MAR |
| Antifungal | CASPOFUNGIN | MAR |
| Antifungal | FLUCONAZOLE | MAR |
| Antifungal | ISAVUCONAZONIUM | MAR |
| Antifungal | ITRACONAZOLE | MAR |
| Antifungal | MICAFUNGIN | MAR |
| Antifungal | POSACONAZOLE | MAR |
| Antifungal | VORICONAZOLE | MAR |
| BL-BLI | AMOXICILLIN/CLAVULANATE | MAR |
| BL-BLI | AMPICILLIN/SULBACTAM | MAR |
| BL-BLI | CEFTAZIDIME/AVIBACTAM | MAR |
| BL-BLI | CEFTOLOZANE/TAZOBACTAM | MAR |
| BL-BLI | PIPERACILLIN/TAZOBACTAM | MAR |
| BL-BLI | TICARCILLIN/CLAVULANATE | MAR |
| Carbapenems | DORIPENEM | MAR |
| Carbapenems | ERTAPENEM | MAR |
| Carbapenems | IMIPENEM/CILASTATIN | MAR |
| Carbapenems | MEROPENEM | MAR |
| ESIBL | AZTREONAM | MAR |
| ESIBL | CEFEPIME | MAR |
| ESIBL | CEFTAROLINE | MAR |
| ESIBL | CEFTAZIDIME | MAR |
| ESIBL | CEFTRIAXONE | MAR |
| Fluoroquinolones | CIPROFLOXACIN | MAR |
| Fluoroquinolones | GEMIFLOXACIN | MAR |
| Fluoroquinolones | LEVOFLOXACIN | MAR |
| Fluoroquinolones | MOXIFLOXACIN | MAR |
| Polymyxins | COLISTIMETHATE | MAR |
| Polymyxins | POLYMYXIN B | MAR |
| Complex Wound Care | SKIN, BACK, RECIPIENT GRAFT SITE PREPARATION,ADDL 100SQCM; DEBRIDEMENT BURN ESCHAR-MAJOR | Operating Room Procedure |
| Complex Wound Care | SKIN, LOWER EXTREMITY, FULL THICKNESS DEBRIDEMENT; SUBCUTANEOUS TISSUE, MUSCLE AND/OR FASCIA | Operating Room Procedure |
| Complex Wound Care | WOUND VAC: NEGATIVE PRESSURE WOUND THERAPY | Procedure |
| Complex Wound Care | IP CONSULT TO WOUND | Procedure |
| Complex Wound Care | OVERLAY MAXXAIR ETS BARIMAXX II BED | Procedure |
| Complex Wound Care | MEASURE WOUND | Procedure |
| Complex Wound Care | WOUND VAC | Procedure |
| Complicated Abdominal pathology | ABDOMEN, LAPAROTOMY, EXPLORATORY, DIAGNOSTIC W/BIOPSY | Operating Room Procedure |
| Complicated Abdominal pathology | US PARACENTESIS | Procedure |
| Tube Feed Related | STOMACH, PERCUTANEOUS ENDOSCOPIC GASTROSTOMY(PEG) | Operating Room Procedure |
| Tube Feed Related | ASPIRATE FEEDING TUBE | Procedure |
| Tube Feed Related | XR ABDOMEN FEEDING TUBE PLACEMENT | Procedure |
| Tube Feed Related | FLUSH FEEDING TUBE | Procedure |
| Tube Feed Related | IP CONSULT TO NUTRITIONAL SUPPORT SURGERY | Procedure |
| Tube Feed Related | DIET TUBE FEEDING CONTINUOUS | Procedure |
| Complicated cardiothoracic pathology | 32100 | CPT |
| Complicated cardiothoracic pathology | 32120 | CPT |
| Complicated cardiothoracic pathology | 32160 | CPT |
| Complicated cardiothoracic pathology | 32220 | CPT |
| Complicated cardiothoracic pathology | 32421 | CPT |
| Complicated cardiothoracic pathology | 32422 | CPT |
| Complicated cardiothoracic pathology | 32480 | CPT |
| Complicated cardiothoracic pathology | 32505 | CPT |
| Complicated cardiothoracic pathology | 32550 | CPT |
| Complicated cardiothoracic pathology | 32551 | CPT |
| Complicated cardiothoracic pathology | 32555 | CPT |
| Complicated cardiothoracic pathology | 32557 | CPT |
| Complicated cardiothoracic pathology | 34.03 | ICD9PCDR |
| Complicated cardiothoracic pathology | 34.06 | ICD9PCDR |
| Complicated cardiothoracic pathology | 34.91 | ICD9PCDR |
| Dialysis | 90935 | CPT |
| Dialysis | C1750 | CPT |
| Dialysis | C1752 | CPT |
| Dialysis | 39.95 | ICD9PCDR |
| Endoscopy | 31510 | CPT |
| Endoscopy | 31525 | CPT |
| Endoscopy | 31526 | CPT |
| Endoscopy | 31570 | CPT |
| Endoscopy | 31575 | CPT |
| Endoscopy | 31579 | CPT |
| Endoscopy | 31615 | CPT |
| Endoscopy | 31620 | CPT |
| Endoscopy | 31622 | CPT |
| Endoscopy | 31623 | CPT |
| Endoscopy | 31624 | CPT |
| Endoscopy | 31625 | CPT |
| Endoscopy | 31628 | CPT |
| Endoscopy | 31629 | CPT |
| Endoscopy | 31634 | CPT |
| Endoscopy | 31645 | CPT |
| Endoscopy | 31646 | CPT |
| Endoscopy | 43200 | CPT |
| Endoscopy | 43202 | CPT |
| Endoscopy | 43205 | CPT |
| Endoscopy | 43215 | CPT |
| Endoscopy | 43219 | CPT |
| Endoscopy | 43220 | CPT |
| Endoscopy | 43231 | CPT |
| Endoscopy | 43232 | CPT |
| Endoscopy | 43234 | CPT |
| Endoscopy | 43235 | CPT |
| Endoscopy | 43236 | CPT |
| Endoscopy | 43238 | CPT |
| Endoscopy | 43239 | CPT |
| Endoscopy | 43240 | CPT |
| Endoscopy | 43241 | CPT |
| Endoscopy | 43242 | CPT |
| Endoscopy | 43244 | CPT |
| Endoscopy | 43245 | CPT |
| Endoscopy | 43247 | CPT |
| Endoscopy | 43248 | CPT |
| Endoscopy | 43249 | CPT |
| Endoscopy | 43255 | CPT |
| Endoscopy | 43256 | CPT |
| Endoscopy | 43259 | CPT |
| Endoscopy | 43260 | CPT |
| Endoscopy | 43261 | CPT |
| Endoscopy | 43262 | CPT |
| Endoscopy | 43264 | CPT |
| Endoscopy | 43265 | CPT |
| Endoscopy | 43268 | CPT |
| Endoscopy | 43269 | CPT |
| Endoscopy | 43271 | CPT |
| Endoscopy | 44360 | CPT |
| Endoscopy | 44372 | CPT |
| Endoscopy | 44373 | CPT |
| Endoscopy | 44388 | CPT |
| Endoscopy | 44389 | CPT |
| Endoscopy | 45300 | CPT |
| Endoscopy | 45330 | CPT |
| Endoscopy | 45331 | CPT |
| Endoscopy | 45378 | CPT |
| Endoscopy | 45379 | CPT |
| Endoscopy | 45380 | CPT |
| Endoscopy | 45381 | CPT |
| Endoscopy | 45382 | CPT |
| Endoscopy | 47556 | CPT |
| Kidney Transplant | 5561 | ICD9PCDR |
| Kidney Transplant | 5569 | ICD9PCDR |
| Liver Transplant | 5051 | ICD9PCDR |
| Liver Transplant | 5059 | ICD9PCDR |
| Mechanical Ventilation | 31500 | CPT |
| Mechanical Ventilation | 31502 | CPT |
| Mechanical Ventilation | 31600 | CPT |
| Mechanical Ventilation | 31603 | CPT |
| Mechanical Ventilation | 31605 | CPT |
| Mechanical Ventilation | 31611 | CPT |
| Mechanical Ventilation | 31720 | CPT |
| Mechanical Ventilation | 94002 | CPT |
| Mechanical Ventilation | 94003 | CPT |
| Transfusion of blood products | 36430 | CPT |
| Transfusion of blood products | 99.04 | ICD9PCDR |
| Transfusion of blood products | 99.05 | ICD9PCDR |
| Transfusion of blood products | 99.07 | ICD9PCDR |
| Transfusion of blood products | 99.09 | ICD9PCDR |
| Urinary catheter | 51701 | CPT |
| Urinary catheter | 51702 | CPT |
| Urinary catheter | 51703 | CPT |
| Urinary catheter | 57.94 | ICD9PCDR |
| Urinary catheter | 57.95 | ICD9PCDR |
| Vascular Access | 36556 | CPT |
| Vascular Access | 36558 | CPT |
| Vascular Access | 36569 | CPT |
| Vascular Access | 38.91 | CPT |
| Vascular Access | 38.93 | CPT |
| Vascular Access | 38.95 | CPT |
| Vascular Access | 38.97 | CPT |
| Vascular Access | 76937 | CPT |
| Vascular Access | 77001 | CPT |
| Vascular Access | C1751 | CPT |

### **Additional details of model selection**

To account for differential effects of any recurring exposure vs no recurring exposure, and of incrementally more exposure, two terms were considered for inclusion in regression models: one representing the effect of any vs no exposure, and another the effect of additional units of exposure above a single exposure. Either, neither, or both terms could be selected.

The stability of the final model was assessed using bootstrap analysis (Stata mfpboot). Two hundred replicate datasets were generated by sampling the original dataset at random with replacement, and the bootstrap inclusion fraction, i.e. the proportion of replicate final models including each variable, calculated.

Supplementary Table 4 Predictions of KPCO acquisition

|  | **Controls (N=5929)** | | **Cases (N=303)** | | **Univariate** | | | **Multivariate  (all variables)** | | | **Final multivariate model** | | | **Bootstraph includsion fraction (%)** |
| --- | --- | --- | --- | --- | --- | --- | --- | --- | --- | --- | --- | --- | --- | --- |
| **Variable** | **n / median** | **% / IQR** | **n / median** | **% / IQR** | **Odds ratio** | **95% Confidence interval** | **p value** | **Odds ratio** | **95% Confidence interval** | **p value** | **Odds ratio** | **95% Confidence interval** | **p value** |  |
| Congestive heart failure | 923 | 15.6% | 49 | 16.2% | 1.05 | (0.76, 1.43) | 0.78 | 1.12 | (0.74, 1.68) | 0.60 |  |  |  | 18.5 |
| Chronic lung disease | 1140 | 19.2% | 50 | 16.5% | 0.83 | (0.61, 1.13) | 0.24 | 0.88 | (0.61, 1.28) | 0.50 |  |  |  | 20.5 |
| Liver disease | 340 | 5.7% | 25 | 8.3% | 1.48 | (0.97, 2.26) | 0.07 | 0.95 | (0.47, 1.93) | 0.89 |  |  |  | 13 |
| Chronic kidney disease | 1087 | 18.3% | 70 | 23.1% | 1.34 | (1.02, 1.76) | 0.04 | 1.32 | (0.87, 2.00) | 0.20 |  |  |  | 32 |
| Metastatic malignancy | 306 | 5.2% | 11 | 3.6% | 0.69 | (0.38, 1.28) | 0.24 | 0.93 | (0.38, 2.27) | 0.88 |  |  |  | 23.5 |
| HIV | 18 | 0.3% | 1 | 0.3% | 1.09 | (0.14, 8.17) | 0.94 | 2.20 | (0.27, 17.99) | 0.46 |  |  |  | 10.5 |
| Diabetes with complication | 502 | 8.5% | 32 | 10.6% | 1.28 | (0.88, 1.86) | 0.21 | 1.27 | (0.75, 2.14) | 0.37 |  |  |  | 20.5 |
| Solid organ transplant | 295 | 5.0% | 24 | 7.9% | 1.64 | (1.07, 2.53) | 0.02 | 0.63 | (0.26, 1.48) | 0.29 |  |  |  | 28 |
| Female | 2636 | 44.5% | 139 | 45.9% | 1.06 | (0.84, 1.33) | 0.63 | 1.21 | (0.94, 1.56) | 0.14 |  |  |  | 36 |
| Department, vs other (reference) |  |  |  |  |  |  |  |  |  |  |  |  |  |  |
| - Other | 3941 | 66.5% | 163 | 53.8% | 1.00 |  |  | 1.00 |  |  |  |  |  |  |
| - STBICU | 595 | 10.0% | 60 | 19.8% | 2.44 | (1.79, 3.32) | <0.001 | 1.26 | (0.79, 2.00) | 0.34 | 1.19 | (0.76, 1.87) | 0.45 | 100 |
| - MICU | 405 | 6.8% | 22 | 7.3% | 1.31 | (0.83, 2.07) | 0.24 | 0.64 | (0.33, 1.23) | 0.18 | 0.61 | (0.32, 1.18) | 0.14 | 100 |
| - LTACH | 988 | 16.7% | 58 | 19.1% | 1.42 | (1.04, 1.93) | 0.03 | 1.56 | (0.62, 3.95) | 0.35 | 1.70 | (0.69, 4.21) | 0.25 | 100 |
| KPCO colonisation pressure (STBICU) | 0 | 0 - 0 | 0 | 0 - 0 | 1.04 | (1.03, 1.05) | <0.001 | 1.01 | (0.99, 1.03) | 0.20 | 1.02 | (1.00, 1.03) | 0.04 | 100 |
| KPCO colonisation pressure (MICU) | 0 | 0 - 0 | 0 | 0 - 0 | 1.02 | (1.00, 1.04) | 0.03 | 1.00 | (0.97, 1.02) | 0.93 | 1.00 | (0.98, 1.03) | 0.94 | 100 |
| KPCO colonisation pressure (LTACH) | 0 | 0 - 0 | 0 | 0 - 0 | 1.00 | (0.99, 1.00) | 0.22 | 1.00 | (0.99, 1.00) | 0.32 | 1.00 | (0.99, 1.00) | 0.26 | 100 |
| KPCO colonisation pressure (Other) | 2 | 0 - 10 | 0 | 0 - 6 | 0.99 | (0.98, 1.00) | 0.06 | 0.99 | (0.97, 1.00) | 0.06 | 0.99 | (0.98, 1.00) | 0.06 | 100 |
| Charlson score | 1 | 0 - 4 | 1 | 0 - 4 | 1.01 | (0.97, 1.05) | 0.73 | 0.96 | (0.87, 1.06) | 0.40 |  |  |  | 25.5 |
| Age | 62 | 50 - 72 | 59 | 49 - 69 | 0.99 | (0.99, 1.00) | 0.06 | 1.00 | (0.99, 1.00) | 0.31 |  |  |  | 28 |
| Acute inpatient days | 12 | 6 - 22 | 19 | 10 - 33 | 1.03 | (1.03, 1.04) | <0.001 | 1.00 | (0.99, 1.02) | 0.71 |  |  |  | 32.5 |
| LTACH inpatient days | 0 | 0 - 0 | 0 | 0 - 0 |  |  |  |  |  |  |  |  |  | 100 |
| (LTACH inpatient days)^-2 |  |  |  |  | 0.86 | (0.84, 0.88) | <0.001 | 0.86 | (0.84, 0.89) | <0.001 | 0.87 | (0.84, 0.89) | <0.001 |  |
| (LTACH inpatient days)^-1 |  |  |  |  | 5.05 | (3.78, 6.75) | <0.001 | 5.20 | (3.70, 7.30) | <0.001 | 5.16 | (3.71, 7.18) | <0.001 |  |
| Mechanical ventilation days | 1 | 0 - 5 | 3 | 0 - 15 | 1.04 | (1.03, 1.05) | <0.001 | 1.03 | (1.00, 1.05) | 0.02 | 1.02 | (1.01, 1.04) | 0.005 | 43.5 |
| Any aminoglycoside | 242 | 4.1% | 19 | 6.3% | 1.57 | (0.97, 2.55) | 0.07 | 0.95 | (0.54, 1.66) | 0.86 |  |  |  | 12.5 |
| Any antifungal | 1168 | 19.7% | 115 | 38.0% | 2.49 | (1.96, 3.17) | <0.001 | 1.01 | (0.66, 1.56) | 0.95 |  |  |  | 35 |
| Antifungal days | 0 | 0 - 0 | 0 | 0 - 6 | 1.09 | (1.07, 1.11) | <0.001 | 1.03 | (0.99, 1.08) | 0.13 |  |  |  | 21 |
| Any beta-lactam/beta-lactamase inhibitor | 1987 | 33.5% | 152 | 50.2% | 2.00 | (1.58, 2.52) | <0.001 | 1.68 | (1.19, 2.37) | 0.003 | 1.69 | (1.28, 2.24) | <0.001 | 83 |
| Beta-lactam/beta-lactamase inhibitor days | 0 | 0 - 3 | 1 | 0 - 6 | 1.07 | (1.04, 1.09) | <0.001 | 0.96 | (0.92, 1.01) | 0.10 |  |  |  | 30 |
| Any carbapenem | 512 | 8.6% | 63 | 20.8% | 2.78 | (2.07, 3.72) | <0.001 | 1.47 | (0.72, 2.99) | 0.29 | 2.56 | (1.59, 4.11) | <0.001 | 54 |
| Carbapenem days | 0 | 0 - 0 | 0 | 0 - 0 | 1.22 | (1.15, 1.29) | <0.001 | 0.99 | (0.83, 1.17) | 0.91 |  |  |  | 16.5 |
| Any complex wound care | 1854 | 31.3% | 136 | 44.9% | 1.79 | (1.42, 2.26) | <0.001 | 1.09 | (0.78, 1.52) | 0.62 |  |  |  | 24 |
| Complex wound care days | 0 | 0 - 1 | 0 | 0 - 2 | 1.27 | (1.17, 1.38) | <0.001 | 1.03 | (0.87, 1.23) | 0.73 |  |  |  | 9 |
| Any complex abdominal pathology | 409 | 6.9% | 43 | 14.2% | 2.23 | (1.59, 3.13) | <0.001 | 1.13 | (0.74, 1.72) | 0.57 |  |  |  | 17 |
| Any complex thoracic pathology | 455 | 7.7% | 51 | 16.8% | 2.43 | (1.78, 3.34) | <0.001 | 1.48 | (1.01, 2.15) | 0.04 | 1.52 | (1.06, 2.19) | 0.02 | 70 |
| Any dialysis | 740 | 12.5% | 104 | 34.3% | 3.66 | (2.86, 4.70) | <0.001 | 2.79 | (1.81, 4.29) | <0.001 | 2.96 | (2.00, 4.39) | <0.001 | 100 |
| Dialysis days | 0 | 0 - 0 | 0 | 0 - 2 | 1.11 | (1.08, 1.14) | <0.001 | 0.93 | (0.87, 0.99) | 0.02 | 0.94 | (0.89, 1.00) | 0.05 | 47.5 |
| Any endoscopy | 963 | 16.2% | 82 | 27.1% | 1.91 | (1.47, 2.49) | <0.001 | 1.24 | (0.91, 1.69) | 0.18 |  |  |  | 38.5 |
| Any extended spectrum cephalosporin | 2633 | 44.4% | 173 | 57.1% | 1.67 | (1.32, 2.10) | <0.001 | 1.29 | (0.93, 1.78) | 0.13 |  |  |  | 57.5 |
| Extended spectrum cephalosporin days | 0 | 0 - 5 | 2 | 0 - 7 | 1.04 | (1.02, 1.06) | <0.001 | 0.97 | (0.94, 1.01) | 0.11 |  |  |  | 56.5 |
| Any fluroquinolone | 1212 | 20.4% | 79 | 26.1% | 1.37 | (1.05, 1.79) | 0.02 | 1.11 | (0.73, 1.69) | 0.63 |  |  |  | 33.5 |
| Fluoroquinolone days | 0 | 0 - 0 | 0 | 0 - 1 | 1.05 | (1.00, 1.10) | 0.08 | 0.96 | (0.88, 1.06) | 0.47 |  |  |  | 29 |
| Liver transplant | 131 | 2.2% | 17 | 5.6% | 2.63 | (1.57, 4.42) | <0.001 | 2.13 | (0.76, 5.99) | 0.15 |  |  |  | 38 |
| Kidney transplant | 45 | 0.8% | 2 | 0.7% | 0.87 | (0.21, 3.60) | 0.85 | 0.77 | (0.16, 3.80) | 0.75 |  |  |  | 19.5 |
| Any transfusion | 2859 | 48.2% | 207 | 68.3% | 2.32 | (1.81, 2.97) | <0.001 | 1.12 | (0.81, 1.57) | 0.49 |  |  |  | 35 |
| Transfusion events | 0 | 0 - 2 | 2 | 0 - 6 | 1.25 | (1.20, 1.30) | <0.001 | 1.07 | (0.99, 1.15) | 0.11 | 1.09 | (1.03, 1.15) | 0.002 | 31 |
| Any enteral feeding | 2792 | 47.1% | 188 | 62.0% | 1.84 | (1.45, 2.33) | <0.001 | 0.99 | (0.71, 1.37) | 0.93 |  |  |  | 25.5 |
| Enteral feeding days | 0 | 0 - 6 | 3 | 0 - 12 | 1.03 | (1.02, 1.04) | <0.001 | 0.99 | (0.96, 1.01) | 0.23 |  |  |  | 37 |
| Any urinary catheter | 948 | 16.0% | 63 | 20.8% | 1.38 | (1.04, 1.84) | 0.03 | 1.16 | (0.80, 1.69) | 0.43 |  |  |  | 28.5 |
| Urinary catheter days | 0 | 0 - 0 | 0 | 0 - 0 | 1.22 | (1.00, 1.49) | 0.04 | 1.36 | (0.72, 2.56) | 0.34 |  |  |  | 39 |
| Any central vascular access | 2708 | 45.7% | 194 | 64.0% | 2.12 | (1.67, 2.69) | <0.001 | 0.81 | (0.58, 1.14) | 0.22 |  |  |  | 38.5 |
| Central vascular access events | 0 | 0 - 1 | 1 | 0 - 3 | 1.57 | (1.45, 1.70) | <0.001 | 1.16 | (0.97, 1.38) | 0.10 |  |  |  | 44.5 |
|  |  |  |  |  |  |  |  |  |  |  |  |  |  |  |
| Any beta-lactam/beta-lactamase inhibitor + Any carbapenem (interaction p=0.006) |  |  |  |  |  |  |  |  |  |  | 1.78 | (1.09, 2.89) | 0.02 |  |

Supplementary Table 5 Predictions of KPCO acquisition including only cases with a prior negative screen

|  | **Final multivariate model including all cases*** | | | **Final multivariate model including only cases with prior negative test (n=208)** | | |
| --- | --- | --- | --- | --- | --- | --- |
| **Variable** | **Odds ratio** | **95% Confidence interval** | **p value** | **Odds ratio** | **95% Confidence interval** | **p value** |
| Department, vs other (reference) |  |  |  |  |  |  |
| - STBICU | 1.19 | (0.76, 1.87) | 0.45 | 1.42 | (0.79, 2.55) | 0.24 |
| - MICU | 0.61 | (0.32, 1.18) | 0.14 | 0.52 | (0.21, 1.29) | 0.16 |
| - LTACH | 1.70 | (0.69, 4.21) | 0.25 | 2.33 | (0.88, 6.19) | 0.09 |
| KPCO colonisation pressure (STBICU) | 1.02 | (1.00, 1.03) | 0.04 | 1.02 | (1.01, 1.04) | 0.006 |
| KPCO colonisation pressure (MICU) | 1.00 | (0.98, 1.03) | 0.94 | 1.02 | (0.99, 1.05) | 0.17 |
| KPCO colonisation pressure (LTACH) | 1.00 | (0.99, 1.00) | 0.26 | 1.00 | (0.99, 1.01) | 0.56 |
| KPCO colonisation pressure (Other) | 0.99 | (0.98, 1.00) | 0.06 | 1.01 | (0.99, 1.02) | 0.32 |
| (LTACH inpatient days)^-2 | 0.87 | (0.84, 0.89) | <0.001 | 0.87 | (0.85, 0.90) | <0.001 |
| (LTACH inpatient days)^-1 | 5.16 | (3.71, 7.18) | <0.001 | 4.57 | (3.21, 6.51) | <0.001 |
| Mechanical ventilation days | 1.02 | (1.01, 1.04) | 0.005 | 1.03 | (1.01, 1.04) | 0.002 |
| Any antifungal |  |  |  | 1.62 | (1.15, 2.29) | 0.006 |
| Any beta-lactam/beta-lactamase inhibitor | 1.69 | (1.28, 2.24) | <0.001 | 1.49 | (1.08, 2.04) | 0.01 |
| Any carbapenem | 2.56 | (1.59, 4.11) | <0.001 |  |  |  |
| Any complex cardiothoracic pathology | 1.52 | (1.06, 2.19) | 0.02 | 1.56 | (1.04, 2.34) | 0.03 |
| Any dialysis | 2.96 | (2.00, 4.39) | <0.001 | 3.48 | (2.22, 5.44) | <0.001 |
| Dialysis days | 0.94 | (0.89, 1.00) | 0.05 | 0.95 | (0.89, 1.01) | 0.10 |
| Any extended spectrum cephalosporin |  |  |  | 1.35 | (0.97, 1.88) | 0.08 |
| Any transfusion |  |  |  | 1.42 | (0.97, 2.07) | 0.07 |
| Transfusion events | 1.09 | (1.03, 1.15) | 0.002 |  |  |  |
| Any beta-lactam/beta-lactamase inhibitor + Any carbapenem | 1.78 | (1.09, 2.89) | 0.02 |  |  |  |

* as shown in Table 1 and Supplementary Table 4

## References

1. Mathers AJ, Poulter M, Dirks D, Carroll J, Sifri CD, Hazen KC. Clinical Microbiology Costs for Methods of Active Surveillance for Klebsiella pneumoniae Carbapenemase-Producing Enterobacteriaceae. Infect Control Hosp Epidemiol **2014**; 35(4): 350-5.

2. Mathers AJ, Carroll J, Sifri CD, Hazen KC. Modified Hodge Test versus Indirect Carbapenemase Test: Prospective Evaluation of a Phenotypic Assay for Detection of Klebsiella pneumoniae Carbapenemase (KPC) in Enterobacteriaceae. J Clin Microbiol **2013**; 51(4): 1291-3.

3. Mathers AJ, Hazen KC, Carroll J, et al. First clinical cases of OXA-48-producing carbapenem-resistant Klebsiella pneumoniae in the United States: the "menace" arrives in the new world. J Clin Microbiol **2013**; 51(2): 680-3.

4. Institute CLaS. Performance Standards for Antimicrobial Susceptibility Testing. m100. Wayne, PA: Clinical Laboratory and Standards Institute, **2017**:251.

5. Clinical, Laboratory, and, Standards, Institute. Performance Standards for Antimicrobial Susceptibility Testing M100-25. M100. Wayne, PA: CLSI, **2015**:238.

6. Hussein K, Sprecher H, Mashiach T, Oren I, Kassis I, Finkelstein R. Carbapenem resistance among Klebsiella pneumoniae isolates: risk factors, molecular characteristics, and susceptibility patterns. Infect Control Hosp Epidemiol **2009**; 30(7): 666-71.

7. Pereira MR, Scully BF, Pouch SM, et al. Risk factors and outcomes of carbapenem-resistant Klebsiella pneumoniae infections in liver transplant recipients. Liver Transpl **2015**; 21(12): 1511-9.

8. O'Horo JC, Farrell A, Sohail MR, Safdar N. Carbapenem-resistant Enterobacteriaceae and endoscopy: An evolving threat. Am J Infect Control **2016**; 44(9): 1032-6.

9. Papadimitriou-Olivgeris M, Marangos M, Fligou F, et al. KPC-producing Klebsiella pneumoniae enteric colonization acquired during intensive care unit stay: the significance of risk factors for its development and its impact on mortality. Diagn Microbiol Infect Dis **2013**; 77(2): 169-73.

10. Jiao Y, Qin Y, Liu J, et al. Risk factors for carbapenem-resistant Klebsiella pneumoniae infection/colonization and predictors of mortality: a retrospective study. Pathog Glob Health **2015**; 109(2): 68-74.

11. Ny P, Nieberg P, Wong-Beringer A. Impact of carbapenem resistance on epidemiology and outcomes of nonbacteremic Klebsiella pneumoniae infections. Am J Infect Control **2015**; 43(10): 1076-80.

12. Bogan C, Kaye KS, Chopra T, et al. Outcomes of carbapenem-resistant Enterobacteriaceae isolation: matched analysis. Am J Infect Control **2014**; 42(6): 612-20.

13. Schwartz-Neiderman A, Braun T, Fallach N, Schwartz D, Carmeli Y, Schechner V. Risk Factors for Carbapenemase-Producing Carbapenem-Resistant Enterobacteriaceae (CP-CRE) Acquisition Among Contacts of Newly Diagnosed CP-CRE Patients. Infect Control Hosp Epidemiol **2016**; 37(10): 1219-25.

14. Nicolas-Chanoine MH, Vigan M, Laouénan C, Robert J, Group” E-cS. Risk factors for carbapenem-resistant Enterobacteriaceae infections: a French case-control-control study. Eur J Clin Microbiol Infect Dis **2019**; 38(2): 383-93.

15. Swaminathan M, Sharma S, Poliansky Blash S, et al. Prevalence and risk factors for acquisition of carbapenem-resistant Enterobacteriaceae in the setting of endemicity. Infect Control Hosp Epidemiol **2013**; 34(8): 809-17.

16. Patel G, Huprikar S, Factor SH, Jenkins SG, Calfee DP. Outcomes of carbapenem-resistant Klebsiella pneumoniae infection and the impact of antimicrobial and adjunctive therapies. Infect Control Hosp Epidemiol **2008**; 29(12): 1099-106.

17. Mariappan S, Sekar U, Kamalanathan A. Carbapenemase-producing Enterobacteriaceae: Risk factors for infection and impact of resistance on outcomes. Int J Appl Basic Med Res **2017**; 7(1): 32-9.

18. Bhargava A, Hayakawa K, Silverman E, et al. Risk factors for colonization due to carbapenem-resistant Enterobacteriaceae among patients exposed to long-term acute care and acute care facilities. Infect Control Hosp Epidemiol **2014**; 35(4): 398-405.

19. Díaz A, Ortiz DC, Trujillo M, Garcés C, Jaimes F, Restrepo AV. Clinical Characteristics of Carbapenem-resistant Klebsiella pneumoniae Infections in Ill and Colonized Children in Colombia. Pediatr Infect Dis J **2016**; 35(3): 237-41.

20. Guh AY, Bulens SN, Mu Y, et al. Epidemiology of Carbapenem-Resistant Enterobacteriaceae in 7 US Communities, 2012-2013. JAMA **2015**; 314(14): 1479-87.

21. Correa L, Martino MD, Siqueira I, et al. A hospital-based matched case-control study to identify clinical outcome and risk factors associated with carbapenem-resistant Klebsiella pneumoniae infection. BMC Infect Dis **2013**; 13: 80.

22. Bleumin D, Cohen MJ, Moranne O, et al. Carbapenem-resistant Klebsiella pneumoniae is associated with poor outcome in hemodialysis patients. J Infect **2012**; 65(4): 318-25.

23. Papadimitriou-Olivgeris M, Marangos M, Fligou F, et al. Risk factors for KPC-producing Klebsiella pneumoniae enteric colonization upon ICU admission. J Antimicrob Chemother **2012**; 67(12): 2976-81.

24. Wiener-Well Y, Rudensky B, Yinnon AM, et al. Carriage rate of carbapenem-resistant Klebsiella pneumoniae in hospitalised patients during a national outbreak. J Hosp Infect **2010**; 74(4): 344-9.

25. Ben-David D, Masarwa S, Navon-Venezia S, et al. Carbapenem-resistant Klebsiella pneumoniae in post-acute-care facilities in Israel. Infect Control Hosp Epidemiol **2011**; 32(9): 845-53.

26. Gasink LB, Edelstein PH, Lautenbach E, Synnestvedt M, Fishman NO. Risk factors and clinical impact of Klebsiella pneumoniae carbapenemase-producing K. pneumoniae. Infect Control Hosp Epidemiol **2009**; 30(12): 1180-5.

27. Torres-Gonzalez P, Cervera-Hernandez ME, Niembro-Ortega MD, et al. Factors Associated to Prevalence and Incidence of Carbapenem-Resistant Enterobacteriaceae Fecal Carriage: A Cohort Study in a Mexican Tertiary Care Hospital. PLoS One **2015**; 10(10): e0139883.

28. Hu Y, Ping Y, Li L, Xu H, Yan X, Dai H. A retrospective study of risk factors for carbapenem-resistant Klebsiella pneumoniae acquisition among ICU patients. J Infect Dev Ctries **2016**; 10(3): 208-13.

29. Dizbay M, Guzel Tunccan O, Karasahin O, Aktas F. Emergence of carbapenem-resistant Klebsiella spp. infections in a Turkish university hospital: epidemiology and risk factors. J Infect Dev Ctries **2014**; 8(1): 44-9.

30. Otter JA, Dyakova E, Bisnauthsing KN, et al. Universal hospital admission screening for carbapenemase-producing organisms in a low-prevalence setting. J Antimicrob Chemother **2016**; 71(12): 3556-61.
